# QT Dispersion Changes after Transcatheter Aortic Valve Implantation in Patients with Aortic Stenosis

**DOI:** 10.21470/1678-9741-2019-0012

**Published:** 2019

**Authors:** Mustafa Zungur

**Affiliations:** 1 Department of Cardiology, Kent Hospital, Izmir, Turkey.

**Keywords:** Aortic Valve, Transcatheter Aortic Valve Replacement, Heart Valve Prosthesis, Echocardiography

## Abstract

**Objective:**

The aim of this study was to evaluate the QT dispersion and echocardiographic parameters in patients before and after transcatheter aortic valve implantation (TAVI).

**Methods:**

One hundred and fifty-two patients with severe aortic valve stenosis (AS) were included in our study. Ninety five patients who underwent aortic valve replacement with TAVI were included in the TAVI group and 57 patients, who refused TAVI, were included in the medical treatment group. The QT interval and echocardiographic parameters of all patients were assessed before and after the procedure (first and sixth months and first year). The QT intervals were taken from the onset of the QRS to the end of the T wave.

**Results:**

All patients had severe AS. The average mean aortic valve gradient was 46.1±12. Left ventricular internal diastolic diameter (LVIDD) and interventricular septum diastolic thickness (IVSDT) did not change signiﬁcantly after TAVI (*P*>0.05). QT dispersion, corrected QT dispersion, and mean aortic valve gradient changed signiﬁcantly six months after TAVI (*P*<0.05). Compared to the medical treatment group, QT dispersion and corrected QT dispersion were significantly decreased at the sixth month in the TAVI group. The incidence of malignant arrhythmias was smaller in the TAVI group than in the medical treatment group. The mortality rate was lower at the first-year follow-up in the TAVI group than in the medical treatment group.

**Conclusion:**

Increased QT dispersion is associated with severe symptomatic AS. After TAVI, QT dispersion reduces.

**Table:** 

Abbreviations, acronyms & symbols				
AS	= Aortic valve stenosis		LVEF	= Left ventricular ejection fraction
ATP	= Adenosine triphosphate		LVH	= Left ventricular hypertrophy
AV	= Atrioventricular		LVIDD	= Left ventricular internal diastolic diameter
AVA	= Aortic valve area		LVMI	= Left ventricular mass index
AVR	= Aortic valve replacement		PCI	= Percutaneous coronary intervention
BMI	= Body mass index		PWTd	= Posterior wall thickness diameter
BNP	= Brain natriuretic peptide		QTcd	= Corrected QT dispersion
CABG	= Coronary artery bypass grafting		QTd	= QT dispersion
DBP	= Diastolic blood pressure		SBP	= Systolic blood pressure
ECG	= Electrocardiogram		SPSS	= Statistical Package for the Social Sciences
EuroSCORE	= European System for Cardiac Operative Risk Evaluation		STS	= Society of Thoracic Surgeons
HR	= Heart rate		TAVI	= Transcatheter aortic valve implantation
IVSDT	= Interventricular septum diastolic thickness		VF	= Ventricular fibrillation
LDL	= Low-density lipoprotein		VT	= Ventricular tachycardia

## INTRODUCTION

Transcatheter aortic valve implantation (TAVI) has recently become an effective therapeutic alternative to surgical treatment for patients with severe symptomatic aortic valve stenosis (AS), particularly for those who were considered as high-risk surgical or inoperable patients. When compared with medical therapy alone, untreated severe AS has a high rate of morbidity and mortality^[1,2]^. TAVI has been suggested to be more reliable in high-risk surgical or inoperable patients in recent studies^[3-6]^.

In patients with severe symptomatic AS, it has been postulated that malignant ventricular arrhythmias play a vital role in the outcomes and sudden cardiac death^[7]^.

QT dispersion (QTd) is the maximum inter-variance between the longest and shortest QT intervals recorded on a standard 12-lead electrocardiogram (ECG). It reflects the homogeneity of myocardial repolarization^[8]^. In addition, QTd can be used as a probable prognostic tool for future ventricular tachyarrhythmias and death^[9,10]^.

Left ventricular hypertrophy (LVH) due to chronic pressure stress is a risk factor for ventricular arrhythmias and sudden cardiac death^[11]^. Increased QTd has been reported in AS patients and has also been shown to be a potential marker for ventricular arrhythmic homogeneity and mortality. QTd reduction by surgical aortic valve replacement (AVR) has also been reported^[8]^. For these reasons, I would like to investigate the effect of TAVI on QTd in patients with severe AS in this study.

## METHODS

### Study Design and Patients

A total of 152 patients with severe AS were followed up prospectively. Ninety-five of these patients underwent AVR with TAVI method. Patients who were found to be suitable for TAVI by the heart team council of our center were included in the TAVI group. Patients who were suitable for surgical AVR were not included in the study. The study was conducted between June 2013 and December 2015. The patients who underwent AVR with TAVI were included in the TAVI group (n=95); the patients who refused TAVI were included in the medical treatment group (n=57). Patients who had atrial fibrillation or flutter, frequent (> 10/min) ventricular extrasystoles, sinus or atrioventricular (AV) node dysfunction, permanent cardiac pacemaker, abnormal serum electrolyte levels, congenital long-QT syndrome, who had been taking any drugs influencing QTd and using antiarrhythmic drugs, with new bundle branch block, or who had become pacemaker dependents after TAVI were excluded. Operative risks for patients were calculated using the Logistic European System for Cardiac Operative Risk Evaluation (EuroSCORE) and the Society of Thoracic Surgeons (STS) Predictive Risk of Mortality scores. Patients with a Logistic EuroSCORE >20% or an STS score >10% were considered as high-risk patients.

Clinical, demographic, 12-lead ECG, and echocardiographic data, procedural variables, and morbidity and mortality rates were recorded for the first and sixth months and the first year following TAVI. First and sixth months and first year follow-up visits after the discharge of patients were performed at our center.

The study was approved by the institutional ethics committee, and all the study-related procedures were performed according to the latest version of the Helsinki Declaration. All patients signed an informed consent form prior to their participation in the study.

### Electrocardiography

Standard 12-lead ECG (25 mm/s) was recorded after a 10-minute rest in the supine position before TAVI and at the first and sixth months after TAVI. QTd calculation was manually performed by two independent cardiologists who were blinded to all patients’ data. The compatibilities of QTd were statistically analyzed. If there was a difference between the results of QTd, the final decision was made by consensus. The QT interval was measured from the onset of the QRS complex to the end of the T wave. The mean of three consecutive interval measurements was used in the analysis. QTd was calculated as the difference between the longest and shortest QT interval measured in each individual ECG lead. QT intervals were corrected with Bazett’s formula (QTc:QT√RR).

### Echocardiography

Patients were evaluated with standard transthoracic M-mode and two-dimensional echocardiographic studies before and after TAVI procedure. Left ventricular diastolic and systolic dimensions and ventricular septal and posterior wall thicknesses were measured at the level of the tips of the mitral valve leaflet. Severe AS was described as a mean aortic valve gradient of ≥ 40 mmHg or an aortic valve area (AVA) of ≤ 1 cm^2^.

### Study Procedures

A mean aortic gradient >40 mmHg, an AVA <1 cm^2^, and a valve area index (valve area/body surface area) <0.6 cm^2^ were considered severe AS^[1]^. Edwards SAPIEN XT valve (Edwards Lifesciences, Irvine, California, USA) balloon-expandable device (n=85) and Medtronic CoreValve (MCV; Medtronic, Minneapolis, Minnesota, USA) self-expandable device (n=10) were used for TAVI. A vascular occlusion device (ProStar XL, Abbott Laboratories, North Chicago, Illinois, USA) was used in eligible patients in terms of femoral artery diameter and anatomy. The surgical cutdown method was applied in patients who were unsuitable for using the iliac and femoral artery vascular closure device. Transesophageal echocardiography and multislice computed tomography were done to determine the diameter of the aortic bioprosthesis. Patients received clopidogrel 75 mg, aspirin 100 mg, and intravenous antibiotherapy before the procedure.

### Statistical Analysis

Continuous variables were expressed as mean ± standard deviation and categorical variables were expressed as percentages. The normal distribution of values was assessed by using the Kolmogorov-Smirnov test and histogram. Paired *t*-test, independent sample t-test, and Wilcoxon-rank test were used for continuous variables, when appropriate. Pearson’s or Spearman’s correlation coefficient were used to assess the relationship between the parameters, when appropriate. A *P*-value < 0.05 represented a statistically significant result. Statistical analysis was carried out using the Statistical Package for the Social Sciences (SPSS) software (SPSS Inc., Chicago, Illinois, USA), version 16.0.

## RESULTS

A total of 152 consecutive patients were enrolled in the study. TAVI was successfully performed in 47 (49.4%) women and 48 (50.6%) men without severe complications during the hospital stay and follow-up period. Demographic characteristics of the study population are presented in Table 1 and basal echocardiographic and electrocardiographic characteristics in Table 2. The baseline demographic characteristics were similar in both groups. There was no difference in baseline echocardiographic, electrocardiographic, and other clinical parameters. No difference was found between these two groups’ in basal QTd, corrected QT dispersion (QTcd), and other electrocardiographic measurements, like heart rate, PR interval, QRS duration, and QRS axis (Table 2). However, TAVI caused a significant reduction in the mean aortic valve gradient, QTd, and QTcd after six months (Table 3). In the sixth month after TAVI, mean aortic gradients, maximum and minimum QT times, QTd, and QTcd time were significantly decreased in the TAVI group, compared to the medical treatment group. Moreover, the QTd significantly decreased in the TAVI group after the TAVI procedure (Table 4). Mortality and arrhythmic complications were found to be smaller in the TAVI group at one-year follow-up than in the medical treatment group (Table 5).

**Table 1:** Demographic and clinical characteristics of patients who underwent TAVI and medical treatment.

	TAVI (n=95)	Medical treatment (n=57)	*P*-value
Age (years)	78.1±7.3	80±6.5	0.675
Male	50(%52.6)	29(%50.8)	0.854
BMI	27.6±5.6	29.4±3.5	0.657
HR	78.3±9.4	80±8.6	0.346
SBP	129±25	135±15	0.435
DBP	82±14	78±22	0.358
Previous CABG	28(%29.4)	18(%31.5)	0.254
Previous PCI	36(%37.8)	21(%36.8)	0.342
LVEF	45.1±10.6	43.2±5.5	0.345
Logistic EuroSCORE	33.4±10.9	35.5±9.4	0.375
STS Score	13.5±6.5	14.5±7.5	0.554
BNP	7793±2450	6850±2850	0.455
LDL	135.4±27.2	142±32.5	0.550
Triglyceride	145.2±35.1	152±25.5	0.650
Hematocrit	36.2±5	34.5±6.5	0.385
Creatine	1.21±0.55	1.42±0.40	0.545

BMI=body mass index; BNP=brain natriuretic peptide; CABG=coronary artery bypass grafting; DBP=diastolic blood pressure; EuroSCORE = European System for Cardiac Operative Risk Evaluation; HR=heart rate; LDL=low-density lipoprotein; LVEF=left ventricular ejection fraction; PCI=percutaneous coronary intervention; SBP=systolic blood pressure; STS=Society of Thoracic Surgeons; TAVI=transcatheter aortic valve implantation

**Table 2:** Basal echocardiographic and electrocardiographic characteristics of patients in TAVI and medical treatment groups.

Parameters	TAVI (n=95)	Medical treatment (n=57)	*P*-value
Heart rate	78.7±9.7	76.1±10.1	0.872
LVIDD	52.1±5.9	50.8±5.1	0.323
IVSDT	13.7±1.5	13.8±1.7	0.860
PWTd	12.7±1.3	12.9±1.1	0.761
LVMI	259.7±12.1	246±15	0.145
Mean aortic gradient	50.1±10.8	49.3±12.5	0.689
Max aortic gradient	86.2±11.5	83.6±14.2	0.370
Max QT	443.7±61.7	445.2±51.8	0.779
Min QT	350.2±30.2	352.3±34.8	0.923
QTd	119.7±24.1	123.5±23.1	0.096
QTcd	136.8±23.3	135.2±20.8	0.679

IVSDT=interventricular septum diastolic thickness; LVIDD=left ventricular internal diastolic diameter; LVMI=left ventricular mass index; PWTd=posterior wall thickness diameter; QTcd=corrected QT dispersion; QTd=QT dispersion; TAVI=transcatheter aortic valve implantation

**Table 3:** Hemodynamic, echocardiographic, and electrocardiographic characteristics of TAVI patients at baseline and the 6^th^ month after TAVI.

Parameters	Before TAVI	6^th ^month after TAVI	*P*-value
SBP	129.2±21.1	127.8±22.6	0.655
DBP	75.4±9.5	79.2±13.8	0.635
Heart rate	78.7±9.7	75.8±11.2	0.285
LVIDD	52.1±5.9	51.3±4.2	0.355
IVSDT	13.7±1.5	12.8±2.2	0.240
PWTd	12.7±1.3	12.3±1.7	0.205
LVMI	259.7±12.1	241±12	0.230
Mean aortic gradient	50.1±10.8	12.7±4.6	<0.001
Max aortic gradient	86.2±11.5	26±5.6	<0.001
Max QT	443.7±61.7	355±45	<0.001
Min QT	350.2±30.2	310±38	<0.001
QTd	119.7±24.1	105±27	<0.001
QTcd	136.8±23.3	110±24	<0.001

DBP=diastolic blood pressure; IVSDT=interventricular septum diastolic thickness; LVIDD=left ventricular internal diastolic diameter; LVMI=left ventricular mass index; PWTd=posterior wall thickness diameter; SBP=systolic blood pressure; QTcd=corrected QT dispersion; QTd=QT dispersion; TAVI=transcatheter aortic valve implantation

**Table 4:** Hemodynamic, echocardiographic, and electrocardiographic characteristics of patients who underwent TAVI and medical treatment at the 6^th^ month.

Parameters	TAVI (at the 6^th^ month)	Medical treatment (at the 6^th^ month)	*P*-value
SBP	127.8±22.6	126.2±15.1	0.470
DBP	79.2±13.8	78.1±12.1	0.580
Heart rate	75.8±11.2	74.2±10.5	0.390
LVIDD	51.3±4.2	50.2±3.8	0.385
IVSDT	12.8±2.2	13.7±2.8	0.375
PWTd	12.3±1.7	13.1±2.5	0.345
LVMI	241±12	248±25	0.350
Mean aortic gradient	12.7±4.6	51.7±11.8	<0.001
Max aortic gradient	26±5.6	86.7±15.5	<0.001
Max QT	355±45	457.3±52.1	<0.001
Min QT	310±38	358.8±31.7	<0.001
QTd	105±27	132.5±28.5	<0.001
QTcd	110±24	141.2±18.5	<0.001

DBP=diastolic blood pressure; IVSDT=interventricular septum diastolic thickness; LVIDD=left ventricular internal diastolic diameter; LVMI=left ventricular mass index; PWTd=posterior wall thickness diameter; SBP=systolic blood pressure; QTcd=corrected QT dispersion; QTd=QT dispersion; TAVI=transcatheter aortic valve implantation

**Table 5:** Mortality and arrhythmia complication rates at the end of one-year follow-up of the patients in the medical treatment group compared with the TAVI group.

Parameters	TAVI (1^st^ year)	Medical treatment (1^st^ year)	*P*-value
Mortality	6 (%6.3)	11 (%19.3)	0.015
Total AV block (permanent or temporary)	9 (%9.5)	6(%10.5)	0.520
Pacemaker implantation	4 (%4.2)	4 (%7.0)	0.346
Ventricular tachycardia	6 (%6.3)	14 (%24.6)	0.002
Ventricular fibrillation	3 (%3.2)	8 (%14.0)	0.016

AV=atrioventricular; TAVI=transcatheter aortic valve implantation

## DISCUSSION

The number of patients with degenerative aortic valve disease is expected to rise in the future. And adults may remain asymptomatic for a long time^[12]^. After heart failure, syncope, and angina, the survival time is shortened^[1]^. Surgical AVR is now the gold standard treatment for patients with severe symptomatic AS. However, some severe symptomatic AS patients may present a very high risk for surgery due to contraindications or comorbidities. They cannot be operated on or are considered high risk for surgery. TAVI is a well-known alternative technique for these patients^[4,13]^.

In patients with symptomatic AS, malignant ventricular arrhythmias play an important role in the development of syncope and sudden cardiac death^[14]^. Some studies have shown that ventricular arrhythmias are more frequent in these patients than in control subjects^[15]^. ECG of the patients before, during, and after syncope were investigated and malignant ventricular arrhythmia was detected during syncope.

QTd, which increases the formation of ventricular arrhythmias, can be calculated from the surface ECG reflecting regional heterogeneity of ventricular repolarization^[16]^. Many disorders, such as ventricular hypertrophy, myocardial ischemia, autonomic neuropathy, electrolyte imbalance, and use of antiarrhythmic drugs, can cause impaired repolarization and increased QTd.

In our study, we investigated the effect of TAVI on QTd in patients with symptomatic severe AS. We found out that QTd decreased in patients with severe AS who underwent TAVI at the sixth postprocedure month.

In our study, six patients (6.3%) died in the TAVI group and 11 (19.3%) patients died in the medical treatment group. One-year mortality was significantly lower in the TAVI group (*P*<0.001) than in the medical treatment group. The ratio of ventricular tachycardia (VT) (*P*<0.001) and ventricular fibrillation (VF) *(P*<0.001) was significantly higher in the medical treatment group than in the TAVI group during Holter monitoring, outpatient visits, and emergency department admissions. In our study, QTd and QTcd time after AVR with TAVI method were significantly shorter than in the medical treatment group.

Arrhythmogenesis depends on the modulation of ion currents, abnormal ventricular structure, and myocardial ischemia^[17]^. Ventricular dilation and fibrosis may affect different regions of the ventricular wall and may cause an increase in the spread of refractory areas. The evidence of increased QTd in patients with hypertrophic cardiomyopathy, acquired forms of long QT syndrome, uremic neuropathy, hypertension in patients with myocardial infarction, and LVH supports the theorem above^[17]^. The effects of AVR on QTd were evaluated by two studies in the literature. Darbar et al.^[18]^ have shown that increased QTd after AVR in patients with significant AS is reduced. This finding was later reported by Orlowska-Baranowska et al.^[19]^. Patients with LVH were also reported to have had myocardial ischemia despite normal coronary angiograms. Thallium scans of patients with significant AS and normal coronary arteries often show perfusion deficits due to microvascular dysfunction^[20]^. Microvascular dysfunction leads to a marked remodeling of the cellular compartments of the myocardium. At the cellular level, studies have shown that adenosine triphosphate (ATP)-sensitive potassium channels are more likely to be opened during ischemia in hypertrophic myocytes compared to normal myocytes. This may prolong the repolarization of the myocardium allowing subsequent depolarisations and triggered activity that initiate ventricular arrhythmias^[21]^.

Another mechanism is the increased myocardial interstitial fibrosis in LVH and it has significant effects on electrical conduction^[22]^. Increased myocardial interstitial fibrosis, intraventricular conduction, and nonhomogeneous repolarization facilitate micro-reentry, and arrhythmia can lead to electrical abnormalities in electrical connections between myocardial fibers^[23]^.

In addition, the cardiac renin-angiotensin-aldosterone system activates the gene expression of collagen and fibronectin. This probably contributes to an increase in total collagen volume in the myocardium^[24]^. On the contrary, AS corrupts coronary blood flow. For this reason, hypertrophy and ischemic heart muscles may cause electrophysiological changes.

Previous studies have shown that heterozygosity in repolarization is a predisposed situation to life-threatening arrhythmias, such as VT and VF. The electrical imbalance of the heart muscle can be detected by QTd using surface ECG.

The recovery of autonomic functions appears to be another way of explaining the effect of TAVI on repolarization markers. Patients with severe AS have reported increased sympathetic nervous system activity and reduced sympathetic baroreflex gain. They also demonstrated normalization of sympathetic nervous system activity and restoration of arterial baroreflex gain after TAVI. Increased sympathetic activity has been shown to increase QT interval. It can be suggested that autonomic dysfunction may increase ventricular repolarization abnormalities and QTd. Mechanical obstruction is significantly reduced after TAVI procedure and cardiac output is increased. And this induces improvement of autonomic dysfunction.

### Study Limitations

Our study has some limitations. The small sample size was the main limitation. Coronary ischemia is improved after TAVI and can reduce fatal arrhythmias, but there was no objective criterion for its distinction; another limitation was that arrhythmia complications in both groups could not be determined exactly. Only in the first and sixth months, patients were evaluated by Holter monitoring. ECG controls were performed only in the third and sixth months and the first year in the outpatient clinic. However, apart from these periods, arrhythmic complications may have occurred.

## CONCLUSION

In our study, postprocedure changes in cardiac conduction times and intervals showed positive changes after TAVI. QTd is one of them. According to the results of our study, QTd is more closely related to mortality and malignant arrhythmias in patients with severe AS treated medically than patients who underwent TAVI. It was determined that mortality and malignant arrhythmia rates were reversed with positive changes in QTd after TAVI.

Long-term recording is therefore better for assessing the incidence of ventricular arrhythmias in these patients. For this reason our results should be confirmed with further studies to determine the effectiveness of TAVI on arrhythmic mortality and morbidity.

**Table:** 

Author's roles & responsibilities	
MZ	Substantial contributions to the conception or design of the work; or the acquisition, analysis, or interpretation of data for the work; drafting the work or revising it critically for important intellectual content; agreement to be accountable for all aspects of the work in ensuring that questions related to the accuracy or integrity of any part of the work are appropriately investigated and resolved; final approval of the version to be published
